# Long-term sleep apnea CPAP via tracheostomy in children with tracheomalacia: 20-year experience

**DOI:** 10.3389/fped.2023.1169613

**Published:** 2023-05-30

**Authors:** Tidarat Sriboonyong, Aroonwan Preutthipan, Malinee Nugboon

**Affiliations:** ^1^ Division of Pediatric Pulmonology, Department of Pediatrics, Faculty of Medicine, Ramathibodi Hospital, Mahidol University, Bangkok, Thailand; ^2^ Nursing Affairs, Faculty of Medicine, Ramathibodi Hospital, Mahidol University, Bangkok, Thailand

**Keywords:** tracheostomy, tracheomalacia, children, continuous positive airway pressure, invasive ventilatory support

## Abstract

**Introduction:**

Children with severe tracheobronchomalacia may need placements of tracheostomies and long-term mechanical ventilation. Due to financial constraints, continuous positive airway pressure (CPAP) machines commonly used to treat obstructive sleep apnea in adults have been utilized to deliver positive distending pressure to such children at our institution for more than 20 years with favorable outcomes. We, therefore, reported our experience with 15 children using this machine.

**Methods:**

This is a retrospective study during 2001–2021.

**Results:**

Fifteen children, 9 boys, aged ranged 3 months–5.6 years, were discharged home with CPAP via tracheostomies. All had co-morbidities including gastroesophageal reflux (*n* = 9, 60%), neuromuscular disorders (*n* = 6, 40%), genetic abnormalities (*n* = 6, 40%), cardiac diseases (*n* = 4, 27%) and chronic lungs (*n* = 3, 20%). Eight (53%) children were aged less than 1 year old. The smallest child was aged 3 months old, weighing 4.9 kg. All caregivers were relatives and non-medical health professionals. The 1-month and 1-year readmission rates were 13% and 66% respectively. No factor-associated unfavorable outcomes were statistically identified. No complications related to CPAP malfunction were found. Five (33%) were weaned off CPAP, and 3 died (2 from sepsis and 1 from a sudden unknown cause).

**Conclusion:**

We first reported the use of sleep apnea CPAP via tracheostomy in children with severe tracheomalacia. In limited-resource countries, this simple device may be another option for long-term invasive ventilatory support. The CPAP use in children with tracheobronchomalacia requires adequately trained caregivers.

## 1. Introduction

Tracheomalacia is a condition of excessive tracheal collapsibility. European Respiratory Society defines tracheomalacia as collapsibility greater than 50% expiratory decline in the cross-sectional luminal area during silent respiration. Either a bronchoscopic or radiological examination can be used to assess the severity of tracheomalacia. Although flexible bronchoscopy is the most often utilized modality, there is no one “gold standard” diagnostic test that is accepted worldwide (1). Mild cases can usually be treated without intervention. However, severe cases can result in life-threatening cyanotic attacks, necessitating immediate intervention to stabilize the airway. Because the causes differ, so as a result, the treatments differ such as surgical stabilization, stabilization with removable stents, and conservative intervention such as continuous positive airway pressure (CPAP) (2). Severe tracheomalacia has been managed with many forms of positive pressure therapy (3) (CPAP, bilevel airway pressure, pressure support, full ventilation). Clinical studies have shown that increasing CPAP with tracheomalacia increases forced expiratory flows at functional residual capacity and it has been suggested that CPAP prevents airway collapse by stenting the airway open (4). To adjust CPAP, bronchoscopy or imaging may be used (1). Severe tracheomalacia with chronic respiratory failure may require a tracheostomy with mechanical ventilator support (1). Some of the children with severe tracheomalacia need long-term mechanical ventilation which is indicated when the patients cannot be weaned from mechanical ventilation.

In developing countries because of limited financial resources for homecare mechanical ventilation, it is difficult to send patients back home with mechanical ventilator support. Consequently, CPAP originally designed as a non-invasive ventilator was intentionally used as an invasive ventilator alternative via tracheostomy.

This retrospective study aims to report our twenty-year experience with fifteen children using this CPAP machine to treat severe tracheobronchomalacia and to identify factors associated with unfavorable outcomes.

## 2. Materials and methods

### 2.1. Study population

This study was approved by the Ethical Clearance Committee on Human Rights Related Research Involving Human Subjects, Faculty of Medicine Ramathibodi Hospital, Mahidol University (COA MURA2021/887). This was a retrospective study of all children with tracheomalacia that were treated with home continuous positive airway pressure via tracheostomy in the Ramathibodi pediatrics home mechanical ventilation program between February 2, 2001, and October 31, 2021. The research followed the international recommendations for Strengthening the Reporting of Observational Studies in Epidemiology (5). The following criteria were required for inclusion: (1) All children with tracheomalacia, aged 0 to 18 years, included in this study, and had a confirmed diagnosis by flexible bronchoscopy in spontaneous breathing, the current gold standard (1); and (2) Children have been treated with CPAP via tracheostomy for more than a year. Children who lost to follow-up were excluded. The age of home CPAP started from the time the patient was first sent home with CPAP. The causes for nonscheduled readmission in 1-year were categorized as respiratory problems (acute lower respiratory tract infection, atelectasis), mechanical CPAP-related problems, and other reasons.

### 2.2. Characteristics of home CPAP via tracheostomy

At our hospital, long-term home mechanical ventilation (HMV) was indicated when the patients could not wean from mechanical ventilation or showed progressive respiratory failure. Tracheostomy with invasive ventilation was used when patients had evidence of inadequate ventilation with non-invasive ventilation, risk of aspiration, dependence on ventilation for more than 16 h, and/or ineffective secretion drainage (3). Depending on their budget, the Philips Respironics REMstar Pro C-Flex, Fisher and Paykel CPAP Machines, and Weinmann CPAP were chosen as CPAP devices. The sleep apnea CPAP was a non-invasive ventilator. We modified CPAP to deliver positive airway pressure through a tracheostomy tube instead of the nasal mask in children. The non-invasive CPAP through tracheostomy system comprises of a CPAP machine, a heated humidifier, a single limb circuit, and a tube that ends at the tracheostomy. To reduce rebreathing and avoid excessive carbon dioxide levels, we connect an exhalation port to the tracheostomy in the single limb circuit closet.

The final decision to initiate HMV was made after a detailed discussion with their parents and multidisciplinary team. The settings of home CPAP were established by bedside titration and monitoring (including clinical symptoms, chest x-ray, laboratory studies, vital signs, and end-tidal carbon dioxide monitoring) that were all within normal range. The discharge occurred when the caregiver's competence was assessed and believed to be safe. The first visit was planned 1–3 months after discharge, depending on the clinical condition of the patient. Successful CPAP weaning was defined as being off from CPAP for 7 consecutive days without concomitant clinical or laboratory signs of chronic ventilatory insufficiency (6).

### 2.3. Data collection

We obtained information on patients using a CPAP machine to treat severe tracheomalacia by retrospectively reviewing medical records. Data included demographic data, baseline clinical characteristics including age, gender, underlying disease, age at initiation of CPAP, age at tracheostomy, and weight. Initial CPAP settings, including duration of CPAP per day, CPAP pressure setting was collected at the time of discharge. Additional data gathered included caregivers, educational level of caregivers, hospital stay before discharge with CPAP, and cuffed tracheostomy tube.

### 2.4. Outcome variable

The primary outcomes were to report twenty-year experience with all children using this CPAP machine to treat severe tracheobronchomalacia that met inclusion and exclusion criteria. Factors including age, weight, underlying disease, CPAP pressure, caregiver, and education of caregivers associated with unfavorable outcomes. Unfavorable outcomes were defined as death or readmission within 6 months after discharge.

### 2.5. Statistical analysis

Statistical analysis was performed using the SPSS (version 22.0) statistical software. Descriptive data were presented as mean ± standard deviation (SD) or median (min, max), depending on normality. Continuous data were compared by using Mann–Whitney tests.

The χ^2^ test was used for categorical data. For all analyzed parameters, *p* < 0.05 was considered statistically significant.

## 3. Results

Out of a total of 15 patients, 9 were boys (60%). All of them used home CPAP *v*ia tracheostomy. Their main underlying disorders were gastroesophageal reflux (*n* = 9, 60%), neuromuscular disease (*n* = 6, 40%), genetic abnormalities (*n* = 6, 40%), cardiac disease (*n* = 4, 27%) and chronic lung disease (*n* = 3, 20%). The patient's characteristics are shown in Table 1. The median age at initiation of home CPAP was 1 year and the median weight was 7.5 kg. The youngest patient was a 3-month-old girl and her body weight was 4.9 kg. The follow-up period for CPAP usage ranged from 2.01–18.77 years. CPAP pressure setting when discharged ranged from 5 to 12 cmH_2_O. Indications for tracheostomy and home CPAP were tracheomalacia and bronchomalacia, 80% and laryngomalacia, 53%. The median length of hospitalization in the critical care unit and ward before home CPAP onset was 101 days (IQR: 23–351). Eighty percent of their caregivers were parents and 67% of caregivers had high school graduate or lower. At discharge, all patients needed 24-hour CPAP support, and of those 4 patients required oxygen supplementation (Table 2). Intermittent weaning from CPAP and oxygen was performed by caregivers as tolerated at home.

**Table 1:** Demographic characteristics.

Clinical characteristics	*N *= 15
Age at initiation of CPAP (years)	1.0 (0.3, 5.6)
Age less than 1 year	8 (53%)
Age at tracheostomy (months)	1.8 (0.3, 4.1)
Sex (male), *n* (%)	9 (60)
Weight (kg)	7.5 (4.9, 12)
Body mass index (kg/m^2^)	17.0 (10, 22)
**Underlying diseases, *n* (%)**	
Neuromuscular disorders	6 (40)
Chronic lungs	3 (20)
Cardiac diseases	4 (27)
Genetic abnormalities	6 (40)
Gastroesophageal reflux	9 (60)
Esophageal atresia with tracheoesophageal fistula	1 (7)
**Locations of airway malacia**	
Laryngomalacia	8 (53)
Tracheomalacia	15 (100)
Bronchomalacia	12 (80)

Values are median (min-max), number (%).

CPAP, continuous positive airway pressure.

**Table 2:** Initial CPAP settings and caregivers.

Initial CPAP settings and caregivers	*N* = 15
CPAP pressure setting when discharged (cmH_2_O)	9 (5, 12)
Duration of CPAP per day (h)	24
Cuffed tracheostomy tube, *n* (%)	5 (33)
Oxygen supplement	4 (26)
**Caregivers, *n* (%)**	
Parents	12 (80)
Relatives	2 (14)
Unrelated	1 (7)
**The educational level of caregivers, *n* (%)**	
Primary school	4 (27)
High school	6 (40)
Bachelor	5 (33)
Hospital stay before discharge with CPAP (days)	101 (23, 351)

Values are median (min-max), number (%).

CPAP, continuous positive airway pressure.

There was a 13% rate of readmission within 1 month after discharge. The 1-year readmission rate was 66%. The most common causes of readmissions in 1-month and 1-year were pneumonia, followed by tracheitis. Five (33%) children have been weaned off their devices. Ten (67%) have been dependent on CPAP. Three (20%) died due to 2 from sepsis and 1 from an unknown cause (Table 3).

**Table 3:** Clinical courses and outcomes of home continuous positive airway pressure.

Outcomes	*N* (%)
Weaned from CPAP	5 (33)
Decannulation of tracheostomy	3 (20)
Remaining on CPAP	10 (67)
Decreasing the duration of CPAP usage	5 (33)
1-month readmission after discharge	2 (13)
Readmission in 1 month due to	2 (13)
Lower respiratory tract infection	
Readmission in 6 months due to	7 (47)
Lower respiratory tract infection	6 (40)
Secretion obstruction causing atelectasis	1 (7)
1-year readmission after discharge	10 (66)
Readmission in 1 year due to	
Lower respiratory tract infection	9 (60)
Secretion obstruction causing atelectasis	1 (7)
Death	3 (20)
At the hospital, sepsis	2 (13)
At home, unknown	1 (7)

CPAP, continuous positive airway pressure.

Bivariable analyses of risk factors found no significant predictor of death or readmission identified any factor whether it was age, weight, underlying diseases, or CPAP pressure itself because our admission rate was very low (Table 4).

**Table 4:** Factors associated with unfavorable outcomes.

Number	Outcomes		*p*-value
	Favorable (*N* = 7)	Unfavorable (*N* = 7)	
Age less than 1 year	3	5	0.315
Male	7	2	0.041
Weight ≤7 kg	2	5	0.132
**Underlying diseases**			
Gastroesophageal reflux	3	6	0.119
Neuromuscular disorders	2	4	0.315
CPAP pressure ≤7 cmH_2_O	2	3	0.608
Cuffed tracheostomy tube	4	1	0.282
Non-parent caregivers	1	2	0.569
Educational ≤ secondary school	4	6	0.282
**Complication**			
≥5 events of lower respiratory tract infection	3	2	1
Granulation tissue formation at tracheostomy	4	4	1

Unfavorable outcomes defined by death or readmission within 6 months after discharge.

CPAP, continuous positive airway pressure.

## 4. Discussion

To the best of our knowledge, this is the first report of using adult sleep apnea CPAP to treat children with severe tracheobronchomalacia via tracheostomy. We had to employ this simple device due to the limited financial resources of our country. In Thailand, home mechanical ventilators are not supported by the government or any health insurance. Therefore, if caregivers/parents would like their children to go home, they have to cover all the expenses of home respiratory care. Otherwise, all these children will have an extended hospital stay under the universal health coverage scheme. The benefits of HMV were shorter hospital stays and lower medical expenses (7–9).

CPAP creates distending pressure that prevents excessive collapse of the tracheal wall during inspiration due to tracheomalacia (10). In this study, for severe tracheomalacia often in combination with bronchomalacia or laryngomalacia, CPAP was applied via tracheostomy to stent the airway. CPAP was used at very young ages, and the youngest patient was a 3-month-old girl, with a body weight of 4.9 kg and there were no complications related to CPAP use. An exhalation port was attached in the single limb circuit closest to the tracheostomy to minimize rebreathing and prevent elevated carbon dioxide. More importantly, this exhalation port also allows excessive inspiratory flow generated from an adult sleep apnea CPAP to be released into the atmosphere. This technique prevents pneumothorax. To ensure safety, a chest x-ray was done to make sure the CPAP setting did not cause overinflation. Heated humidification systems were connected to the inspiratory circuit to prevent dry airways and oxygen source can be connected to a heated humidified circuit.

CPAP pressure setting when discharged ranged from 5 to 12 cmH_2_O which were established by bedside titration and cardiopulmonary monitoring (including chest wall movement, chest x-ray, pulse oximeter, vital signs, and capnometry and machine output). An official American Thoracic Society Clinical Practice Guideline: Pediatric Chronic Home Invasive Ventilation, and professional in-home caregivers (e.g., nurses) as required to support the family were arranged before discharge or at least two family caregivers should have had specialized training for the child's care (11). Eighty percent of caregivers were parents and 67% of caregivers were only high school graduates or lower, more than the findings of Kim, Hyang Sook et al. (12). Therefore, even with a lack of well-trained nursing staff, HMV education for caregivers was successful. This comprehensive HMV training program was done by a multidisciplinary team comprising physicians, nurses, and social workers. The program took roughly a month to complete before discharge. Checklists were provided for the HMV training program.

Tracheomalacia's symptoms typically improve or resolve over time because the child grows, and the tracheal cartilage strengthens and stiffens (2). In our study, the 1-month readmission rate was 13% from pneumonia. The rate of nonscheduled hospital admission was lower in our study. Sheila S et al. (13), in their study consisting of 19 children with home mechanical ventilators via tracheostomy, determined the incidence of hospital admissions in the first month after discharge as 42%, and pneumonia and tracheitis were the most common reasons for admission. In Gizem et al.'s (14), study of 70 children with invasive HMV, 30% of them were readmitted to the hospital within 1 month mostly because of tracheostomy-related complications. Thirteen percent of children died at our hospital due to sepsis and 7% died at home with unknown causes. There was no complication related to CPAP use. Compared to the literature review from Edwards JD, et al.*,* 47 of 228 children on chronic positive-pressure ventilation via tracheostomy died over 22 years. They found the commonest cause of mortality was a progression of the reason for chronic respiratory failure or underlying condition (34%) (15).

In limited-resource countries, this sleep apnea CPAP, which was initially intended to be a non-invasive ventilator, was used for long-term invasive ventilatory support via tracheostomy. The cost of a CPAP machine and heated humidified is around 1,000 US dollars which is much cheaper than a regular home mechanical ventilator approximately one-third of a conventional home ventilator. More importantly, no CPAP-related problems occurred over the 20-year experience of 15 patients.

This study has several limitations. The first limitations are the small sample and heterogeneity by age and co-morbidities, which may not have sufficient power to identify risk factors for poor outcomes and prevent readmission and may be selection bias. Second, the study was conducted at a single center and may reflect center-specific procedures for managing CPAP via tracheostomy, an HMV training program, and follow-up care. Third, since this was a retrospective study, we were unable to control for several confounding factors, including caregiver knowledge, home environment, and family members, which might have affected readmission outcomes. Lastly, this study focused only on death or readmission. Future studies should consider multicenter studies and investigate the caregiver's HMV skill, quality of care, and quality of life for both the patient and the caregiver.

## 5. Conclusion

In conclusion, a non-invasive CPAP ventilator via tracheostomy in children with tracheomalacia may be safely used as a substitute for an invasive long-term home mechanical ventilator in a country with limited resources. The CPAP use in children with tracheobronchomalacia requires adequately trained caregivers.

## Data Availability

The raw data supporting the conclusions of this article will be made available by the authors, without undue reservation.

## References

[1] Wallis C Alexopoulou E Antón-Pacheco JL Bhatt JM Bush A Chang AB ERS Statement on tracheomalacia and bronchomalacia in children. Eur Respir J. (2019) 54(3):6–9. 10.1183/13993003.00382-2019 31320455

[2] Carden KA Boiselle PM Waltz DA Ernst A. Tracheomalacia and tracheobronchomalacia in children and adults: an in-depth review. Chest. (2005) 127(3):984–1005. 10.1378/chest.127.3.984 15764786

[3] Preutthipan A. Home mechanical ventilation in children. Indian J Pediatr. (2015) 82(9):852–9. 10.1007/s12098-015-1842-z 26223874

[4] Davis S Jones M Kisling J Angelicchio C Tepper RS. Effect of continuous positive airway pressure on forced expiratory flows in infants with tracheomalacia. Am J Respir Crit Care Med. (1998) 158(1):148–52. 10.1164/ajrccm.158.1.9711034 9655721

[5] von Elm E Altman DG Egger M Pocock SJ Gøtzsche PC Vandenbroucke JP. The strengthening the reporting of observational studies in epidemiology (STROBE) statement: guidelines for reporting observational studies. J Clin Epidemiol. (2008) 61(4):344–9. 10.1016/j.jclinepi.2007.11.008 18313558

[6] Ghiani A Paderewska J Sainis A Crispin A Walcher S Neurohr C. Variables predicting weaning outcome in prolonged mechanically ventilated tracheotomized patients: a retrospective study. J Intensive Care. (2020) 8:19. 10.1186/s40560-020-00437-4 32123565 PMC7035768

[7] Preutthipan A Nugboon M Chaisupamongkollarp T Kuptanon T Kamalaporn H Leejakpai A. An economic approach for children with chronic ventilation support. Curr Pediatr Rep. (2014) 2(1):1–8. 10.1007/s40124-013-0038-0

[8] Noyes J Godfrey C Beecham J. Resource use and service costs for ventilator-dependent children and young people in the UK. Health Soc Care Community. (2006) 14(6):508–22. 10.1111/j.1365-2524.2006.00639.x 17059493

[9] Saiphoklang N Kanitsap A Ruchiwit P Pirompanich P Sricharoenchai T Cooper CB. Patient characteristics and outcomes of a home mechanical ventilation program in a developing country. Lung India. (2019) 36(3):207–11. 10.4103/lungindia.lungindia_219_18 31031340 PMC6503720

[10] Hysinger EB. Laryngomalacia, tracheomalacia and bronchomalacia. Curr Probl Pediatr Adolesc Health Care. (2018) 48(4):113–8. 10.1016/j.cppeds.2018.03.002 29622320

[11] Sterni LM Collaco JM Baker CD Carroll JL Sharma GD Brozek JL An official American thoracic society clinical practice guideline: pediatric chronic home invasive ventilation. Am J Respir Crit Care Med. (2016) 193(8):e16–35. 10.1164/rccm.201602-0276ST 27082538 PMC5439679

[12] Kim HS Lee CE Yang YS. Factors associated with caring behaviors of family caregivers for patients receiving home mechanical ventilation with tracheostomy: a cross-sectional study. PLoS One. (2021) 16(7):e0254987. 10.1371/journal.pone.0254987 34288975 PMC8294500

[13] Kun SS Edwards JD Ward SL Keens TG. Hospital readmissions for newly discharged pediatric home mechanical ventilation patients. Pediatr Pulmonol. (2012) 47(4):409–14. 10.1002/ppul.21536 21901855 PMC3694986

[14] Özcan G Zirek F Tekin MN Bakirarar B Çobanoğlu N. Risk factors for first nonscheduled hospital admissions of pediatric patients on home mechanical ventilation. Pediatr Pulmonol. (2021) 56(10):3374–9. 10.1002/ppul.25581 34297898

[15] Edwards JD Kun SS Keens TG. Outcomes and causes of death in children on home mechanical ventilation via tracheostomy: an institutional and literature review. J Pediatr. (2010) 157(6):955–9.e2. 10.1016/j.jpeds.2010.06.012 20713294

